# Models of Collisionless Quasineutral Solar Wind Current Sheets

**DOI:** 10.1007/s11207-025-02551-8

**Published:** 2025-10-13

**Authors:** Sophie Boswell, Thomas Neukirch, Anton Artemyev, Ivan Vasko, Oliver Allanson

**Affiliations:** 1 School of Mathematics and Statistics, University of St. Andrews, St. Andrews, UK; 2 Department of Earth, Planetary, and Space Sciences, University of California, Los Angeles, Los Angeles, CA USA; 3 William B. Hanson Center for Space Sciences, University of Texas at Dallas, Richardson, TX USA; 4 Space Environment and Radio Engineering, School of Engineering, University of Birmingham, Birmingham, UK; 5 Department of Earth and Environmental Sciences, University of Exeter, Penryn, UK; 6 Department of Mathematics and Statistics, University of Exeter, Exeter, UK

**Keywords:** Electric currents and current sheets, Solar wind, theory, Plasma theory, Magnetic fields, models

## Abstract

In situ measurements of kinetic scale current sheets in the solar wind show that they are often approximately force-free although the plasma $\beta$ is of order one. They frequently display systematic asymmetric and anti-correlated spatial variations of their particle density and temperature across the current sheet, leaving the plasma pressure essentially uniform. These observations of asymmetries have previously been modelled theoretically by adding additional terms to both the ion and electron distribution functions of self-consistent force-free collisionless current sheet models with constant density and temperature profiles. In this article we present the results of a modification of these models in which only the electron distribution function has an additional term, whereas the ion distribution function is kept as a thermal (Maxwellian) distribution function. In this case the nonlinear quasineutrality condition no longer has a simple analytical solution and therefore has to be solved alongside Ampère’s law. We find that while the magnetic field remains approximately force-free, the non-zero quasineutral electric field gives rise to an additional spatial substructure of the plasma density inside the current sheet. We briefly discuss the potential relation between our theoretical findings and current sheet observations.

## Introduction

Current sheets are ubiquitous features of space and astrophysical plasmas. They play a fundamental role in many activity processes, e.g. magnetic reconnection. The present study is motivated by kinetic scale current sheets observed in the solar wind throughout the inner heliosphere (e.g. Vasko et al. 2021, 2022; Lotekar et al. 2022).

We particularly focus on one-dimensional models of currents sheets for which the magnetic field component perpendicular to the current sheet vanishes (“tangential discontinuities” as opposed to “rotational discontinuities” for which the perpendicular magnetic field component is non-zero). We note that even if the perpendicular magnetic field component is non-zero, it is often so small compared to the magnetic field magnitude within the current sheet that it cannot be determined reliably even using multi-spacecraft observations (see, e.g. Knetter et al. 2004; Wang et al. 2024). In situ measurements show that these current sheets are often approximately one-dimensional and force-free, i.e. that the current density is aligned with the magnetic field. This implies that the magnitude of the magnetic field remains approximately constant while the magnetic field direction rotates across the current sheet (e.g Burlaga, Lemaire, and Turner 1977; Lepping and Behannon 1986; Neugebauer 2006; Paschmann et al. 2013).

Statistical analysis of a large number of current sheet observations (Artemyev et al. 2019; Artemyev, Angelopoulos, and Vasko 2019) recently revealed systematic anticorrelated spatial variations of the plasma density, $n$, and the ion and electron temperatures, $T_{i,e}$, across the current sheet, leaving the plasma pressure uniform. Neukirch et al. (2020) presented self-consistent collisionless equilibrium models for current sheets with the observed properties. These models were based on a modification of the equilibrium distribution functions for the force-free Harris sheet (Harrison and Neukirch 2009a; Neukirch, Wilson, and Harrison 2009). Whereas in the usual force-free case the ion distribution function can be taken to be simply a thermal (Maxwellian) distribution, for mathematical convenience Neukirch et al. (2020) had to modify both the electron and the ion distribution functions. In this article we investigate the consequences of leaving the ion distribution function unchanged from the force-free case and modifying only the electron distribution function.

The article is structured as follows. In Section 2, we will give a brief overview of the general theory of the collisionless force-free Harris sheet and the modifications made to the distribution functions by Neukirch et al. (2020). In Section 3, we present how the mathematical problem of finding current sheet solutions changes if only the electron distribution function is modified, in particular the resulting nonlinear quasineutrality condition linking the electric potential and the magnetic vector potential. We then show how one can find solutions by different methods, including solving the full nonlinear problem with numerical methods. In Section 4, we present a summary and our conclusions.

## Background Theory

We briefly summarise the background theory of one-dimensional collisionless force-free current sheets in this section (for a more detailed account see, e.g. Neukirch, Wilson, and Allanson 2018). We use Cartesian coordinates $x$, $y$, $z$ and make the choice that spatial variations occur in the $z$-direction only. We investigate current sheets that only have non-vanishing magnetic field components in the $x$- and $y$-directions, i.e. $\mathbf{B}(z)= [B_{x}(z), B_{y}(z), 0]$. Solutions to the stationary Vlasov equation, i.e. equilibrium distribution functions, only depend on constants of motion related to the charged particle dynamics in the electromagnetic fields (see, e.g. Schindler 2006, Chapter 6). For the chosen set-up the three known constants of motion are associated with the symmetries of the problem, i.e. time-invariance, translational-invariance, and are

- the Hamiltonian (total energy) for the particle species, $s$, given by $H_{s}=m_{s}v^{2}/2 + q_{s}\Phi $, and
- the $x$- and $y$-components of the canonical momentum, $p_{x,s} = m_{s}v_{x} + q_{s}A_{x}$ and $p_{y,s}=m_{s}v_{y} + q_{s}A_{y}$.

Here, $v_{x}$, $v_{y}$, and $v_{z}$ are the $x$-, $y$-, and $z$-components of the velocity, and $v^{2}=v_{x}^{2}+v_{y}^{2}+v_{z}^{2}$. Also, $\Phi (z)$ is the electric potential, $A_{x}(z)$ and $A_{y}(z)$ are the $x$- and $y$-components of the vector potential, and $m_{s}$ and $q_{s}$ are the mass and electric charge of particle species $s$, respectively. In this article we assume that the plasma consists only of electrons ($s=e$) and protons ($s=i$).

Self-consistent solutions to the Vlasov-Maxwell equations are found by calculating the charge and current densities from the equilibrium distribution functions $F_{s}(H_{s}, p_{x,s}, p_{y,s})$, and using them as source terms in the inhomogeneous Maxwell equations. The homogeneous Maxwell equations (Faraday’s law and the solenoidal condition for the magnetic field) are automatically satisfied by the fact that the electromagnetic fields are time-independent and the use of the electric and vector potentials. For quasineutral plasmas, Gauss’s law for the electric potential is well approximated by the quasineutral condition

1 $$ \sigma (\Phi ,A_{x},A_{y}) = e[n_{i}(\Phi ,A_{x},A_{y}) - n_{e}(\Phi ,A_{x},A_{y})] =0 , $$

where $\sigma $ is the charge density, $e$ is the elementary charge, and $n_{i}$ and $n_{e}$ are the proton and electron particle densities defined by the zeroth order velocity moments of the distribution functions,

2 $$ n_{s} = \int F_{s}(H_{s}, p_{x,s}, p_{y,s})\, \mathrm{d}^{3} v. $$

This is coupled with Ampère’s law for the vector potential,

3 $$\begin{aligned} -\frac{\mathrm{d}^{2} A_{x}}{\mathrm{d} z^{2}} =& \mu _{0} j_{x}( \Phi ,A_{x},A_{y}), \end{aligned}$$

4 $$\begin{aligned} - \frac{\mathrm{d}^{2} A_{y}}{\mathrm{d} z^{2}} =& \mu _{0} j_{y}( \Phi ,A_{x},A_{y}) , \end{aligned}$$

where $\mu _{0}$ is the permeability of free space and the current density components are defined by

5 $$ j_{k} (\Phi ,A_{x},A_{y}) = \sum _{s} q_{s} \int v_{k} \,F_{s}(H_{s}, p_{x,s}, p_{y,s})\, \mathrm{d}^{3} v, \quad k=x, y. $$

The model we use as a starting point for our investigations is the force-free Harris sheet for which a self-consistent distribution function was first found by Harrison and Neukirch (2009a). The magnetic field is given by

6 $$ \mathbf{B}(z)=B_{0} \left [\tanh (z/L),\frac{1}{\cosh (z/L)},0\right ] , $$

such that $B_{x}^{2} + B_{y}^{2} = B_{0}^{2} = const$. Here, $B_{0}$ is the magnitude of the magnetic field and $L$ is the typical current sheet width.

Electron and ion distribution functions self-consistent with this magnetic field profile are given by (see e.g. Harrison and Neukirch 2009a; Neukirch et al. 2020)

7 $$\begin{aligned} F_{e}(H_{e},p_{x,e},p_{y,e}) =& \frac{n_{0}}{\sqrt{2\pi v_{th,e}^{2}}^{3}} \exp (-\beta _{e}H_{e}) \times \\ & \; \left [ -\frac{1}{2}\cos (\beta _{e}u_{0}p_{x,e}) \exp \left ( \frac{u_{0}^{2}}{2 v_{th,e}^{2}}\right ) + \right . \\ & \qquad \; \left . \exp (\beta _{e}u_{0}p_{y,e}) \exp \left (- \frac{u_{0}^{2}}{2 v_{th,e} ^{2}}\right ) +b \right ], \end{aligned}$$

8 $$\begin{aligned} F_{i}(H_{i},p_{x,i},p_{y,i}) =& \frac{n_{0,i}}{\sqrt{2\pi v_{th,i}^{2}}^{3}}\exp (-\beta _{i}H_{i}), \end{aligned}$$

where $n_{0}$ and $n_{0,i}$ are typical particle densities, $u_{0}$ is a constant velocity parameter, $b$ is a dimensionless parameter with $b+\frac{1}{2}$ being the value of the plasma $\beta$, and the usual notation of $\beta _{s}=(k_{B}T_{s})^{-1}$ for the inverse temperature has been used, with $k_{B}$ the Boltzmann constant. Also, $v_{th,s}^{2} = (m_{s}\beta _{s})^{-1}$ is the usual thermal velocity of particle species $s$.

For completeness and later reference we also note that for this current sheet equilibrium one finds that the electric potential $\Phi (z)$ is identically zero, and the vector potential components $A_{x}(z)$ and $A_{y}(z)$ are given by

9 $$\begin{aligned} A_{x,ff}(z) = &B_{0}L\arctan [\sinh (z/L)], \end{aligned}$$

10 $$\begin{aligned} A_{y,ff}(z) = & -B_{0}L\ln [\cosh (z/L)]. \end{aligned}$$

We remark that the only gauge transformation allowed for the one-dimensional current sheets we investigate in this article, compatible with the symmetry properties we use, is additive constants to the electric and vector potentials. We fix the gauge by assuming that $A_{x,ff}(z)$ is an odd function of $z$ and $A_{y,ff}(z)$ is an even function of $z$ with $A_{y,ff}(0)=0$. More details regarding this current sheet model and the modification that will be described next can be found in Appendix A.

To be able to model the observed spatial asymmetries in the electron and ion density and temperature (Artemyev et al. 2019; Artemyev, Angelopoulos, and Vasko 2019) Neukirch et al. (2020) added the following additional term to both the ion and electron distribution functions

11 $$ \Delta F_{s}= \delta n_{s} \left ( \frac{\kappa _{s}}{2\pi v_{th,s}^{2}} \right )^{\frac{3}{2}}\beta _{s}u_{0}p_{x,s}\left ( \frac{5}{2}-\kappa _{s}\beta _{s}H_{s}\right ) e^{-\kappa _{s}\beta _{s}H_{s}}, $$

where $\delta n_{s}$ is a constant with the dimension of a particle density, and $\kappa _{s}$ is a dimensionless parameter.

The magnetic field remains as given in Equation 6, i.e. force-free, and so the force-free condition detailed in Harrison and Neukirch (2009b) that requires the relevant component of the pressure tensor, in this case the $zz-$ component, ${P}_{zz}$, to be constant across the sheet is upheld (see Appendix A for details). However, the additional term in the distribution functions leads to an asymmetric contribution to the particle density of the form (Neukirch et al. 2020)

12 $$ \Delta n_{s} = \epsilon n_{0}\frac{2A_{x,ff}}{B_{0}L} $$

where $\delta n_{e} = - \delta n_{i} (\beta _{i}/\beta _{e}) =\epsilon n_{0}$. In combination with a constant pressure, the non-uniform plasma density corresponds to an anti-correlated temperature asymmetry across the sheet.

One interesting point to note is that the ion distribution function for the force-free Harris sheet with constant density and temperature can be chosen to simply be a thermal distribution function (Maxwellian). However, Neukirch et al. (2020) had to modify the ion distribution function in the same way as the electron distribution function in order to have $\Phi =0 $ as an exact solution of the quasineutrality condition, and to leave the magnetic field structure unchanged (see Appendix A). In this article we will investigate how the solution changes if one assumes that the ion distribution function is kept as a purely thermal distribution.

## Quasineutral Theory

### Basic Equations

When using the thermal ion distribution function given by Equation 8 together with the full electron distribution function given by the sum of Equation 7 and the additional population given by Equation 11, getting a self-consistent solution becomes more complicated and generally requires the use of numerical methods, as we will explain below. The quasineutrality condition takes the form

13 $$ n_{0,i}e^{-e\beta _{i}\Phi} - n_{0} \left [ e^{e\beta _{e}\Phi} N(A_{x},A_{y}) -\epsilon e\beta _{e}u_{0}A_{x}(1 + \kappa _{e}e\beta _{e}\Phi )e^{ \kappa _{e}e\beta _{e}\Phi} \right ] = 0, $$

where

14 $$ N(A_{x},A_{y}) = b - \frac{1}{2}\cos (e\beta _{e}u_{0}A_{x})+ e^{-e\beta _{e}u_{0}A_{y}}. $$

In Equation 13, we have used $q_{i} = e$ and $q_{e}=-e$.

The quasineutrality condition is coupled with Ampère’s law

15 $$\begin{aligned} -\frac{d^{2}A_{x}}{dz^{2}} =& \mu _{0}en_{0}u_{0}e^{e\beta _{e}\Phi} \frac{1}{2}\sin (e\beta _{e}u_{0}A_{x}) - \epsilon \mu _{0}en_{0}u_{0}e \beta _{e}\Phi e^{\kappa _{e}e\beta _{e}\Phi}, \end{aligned}$$

16 $$\begin{aligned} -\frac{d^{2}A_{y}}{dz^{2}} =& -\mu _{0}en_{0}u_{0}e^{e\beta _{e}\Phi}e^{-e \beta _{e}u_{0}A_{y}}, \end{aligned}$$

defining a differential-algebraic (DAE) system of equations for $\Phi $, $A_{x}$, and $A_{y}$. For $\epsilon = 0$, we obtain the force-free case where $\Phi =0$, $A_{x}=A_{x,ff}$, and $A_{y}=A_{y,ff}$ (Appendix A). The various parameters satisfy the relations (see, e.g. Neukirch, Wilson, and Allanson 2018; Neukirch et al. 2020)

17 $$\begin{aligned} N(A_{x,ff},A_{y,ff}) =& b + \frac{1}{2}, \end{aligned}$$

18 $$\begin{aligned} n_{0,i} =& n_{0}\left (b+\frac{1}{2}\right ), \end{aligned}$$

19 $$\begin{aligned} \frac{B_{0}L}{2} =& -\frac{1}{e\beta _{e}u_{0}}, \end{aligned}$$

20 $$\begin{aligned} \frac{B_{0}}{L} =& -\mu _{0}en_{0}u_{0}, \end{aligned}$$

21 $$\begin{aligned} \frac{B_{0}^{2}}{2\mu _{0}} =& \frac{n_{0}}{\beta _{e}}, \end{aligned}$$

22 $$\begin{aligned} L^{2} =& \frac{2}{\mu _{0}e^{2}\beta _{e}n_{0}u_{0}^{2}}. \end{aligned}$$

Equation 13 is not solved by $\Phi =0$ and the force-free vector potential components $A_{x,ff}$ and $A_{y,ff}$. This can, for example, be seen by considering the final term, which depends linearly on $A_{x}$ and, therefore, would still vary with $z$ when $\Phi =0$. Therefore, to get self-consistent solutions for the electric potential and the magnetic field, the set of Equations 13 – 16 have to be solved either numerically or using analytical approximations.

### Expanding the Electric Potential for Small $\epsilon $

As noted in Neukirch et al. (2020), the value of the parameter $\epsilon $ needed for the theoretical density asymmetry to match the observed level of asymmetry is quite small ($\epsilon \approx 0.05$). This suggests that a method to obtain sufficiently accurate approximate solutions could be based on an expansion in $\epsilon $ of the problem defined by Equations 13 – 16.

Neukirch et al. (2022) expanded the electric potential about $\Phi = 0$, such that $\Phi = \epsilon \Phi _{1} + \cdots$ to obtain an approximate solution of Equation 13 up to order $\epsilon ^{1}$. Under the assumption that the magnetic field, and hence magnetic vector potential, remain unchanged, one obtains the following relation between the electric potential and the (force-free) vector potential:

23 $$ e\beta _{e}\Phi _{1} = - \frac{2}{\left (b + \frac{1}{2}\right )\left (1 + \frac{\beta _{i}}{\beta _{e}}\right )} \frac{A_{x,ff}}{B_{0}L}. $$

When substituted into the corresponding expression for the asymmetric electron density term $\Delta n_{e}$ this results in

24 $$ \Delta n_{e} = \epsilon n_{0} \frac{\frac{\beta{i}}{\beta _{e}}}{\left (1 +\frac{\beta{i}}{\beta _{e}}\right )} \frac{2A_{x,ff}}{B_{0}L} . $$

Comparing Equation 24 with Equation 12 one notices that they differ by the factor $\frac{\frac{\beta{i}}{\beta _{e}}}{1 +\frac{\beta{i}}{\beta _{e}}}$ in the approximate expression for the quasineutral case. For $\beta _{i}=\beta _{e}$, for example, this implies that for this level of approximation the asymmetric density component would differ from that in Equation 12 by a factor $1/2$.

However, from Ampère’s law (Equations 15 and 16) it is clear that the assumption that the magnetic field remains unchanged at order $\epsilon ^{1}$ is inconsistent, because the current density depends on the electric potential $\Phi $. Hence, it will be modified for $\Phi \ne 0$ and the vector potential for the force-free magnetic field will no longer be an exact solution of Ampère’s law. One therefore has to improve this “crude” approximation by extending the expansion to include the vector potential components, which will be done in the next subsections.

The reason we include the “crude” approximation presented in this subsection is that the resulting particle densities and temperatures have the same spatial structure as those found by Neukirch et al. (2020), albeit scaled by a constant factor. This allows us to illustrate the difference between the spatial variation of the original model and the more consistent solutions that will be presented in the next two subsections. The “crude” approximation results are therefore included in Figures 2 and 4 for comparison.

### Full Expansion Method for Small $\epsilon $: Linear Case

We now generalise the expansion method of Neukirch et al. (2022) to include the magnetic vector potential components $A_{x}$ and $A_{y}$. In normalised form, with $\bar{z} = \frac{z}{L}$, $\bar{\Phi}=e\beta _{e}\Phi $, and all vector potential components normalised by $B_{0}L$, Equations 13 – 16 become

25 $$\begin{aligned} 0 =& \left (b+\frac{1}{2}\right )e^{-\frac{\beta _{i}}{\beta _{e}} \bar{\Phi}}-e^{\bar{\Phi}}N(\bar{A}_{x},\bar{A}_{y}) -2\epsilon (1+ \kappa _{e}\bar{\Phi})e^{\kappa _{e}\bar{\Phi}}\bar{A}_{x}, \end{aligned}$$

26 $$\begin{aligned} N(\bar{A}_{x},\bar{A}_{y}) =&b-\frac{1}{2}\cos (2\bar{A}_{x})+e^{2 \bar{A}_{y}}, \end{aligned}$$

27 $$\begin{aligned} \frac{d^{2}\bar{A}_{x}}{d\bar{z}^{2}} =& -\frac{1}{2}e^{\bar{\Phi}} \sin (2\bar{A}_{x}) -\epsilon \bar{\Phi}e^{\kappa _{e}\bar{\Phi}}, \end{aligned}$$

28 $$\begin{aligned} \frac{d^{2}\bar{A}_{y}}{d\bar{z}^{2}} =& -e^{\bar{\Phi}}e^{2\bar{A}_{y}}. \end{aligned}$$

As in Section 3.2, the electric potential is expanded about $\bar{\Phi} = 0$. The magnetic vector potential components are expanded about their force-free solutions $\bar{A}_{x,ff}$ and $\bar{A}_{y,ff}$, such that $\bar{A_{x}} = \bar{A}_{x,ff} + \epsilon \bar{A}_{x1} +\cdots$ and $\bar{A}_{y} = \bar{A}_{y,ff} + \epsilon \bar{A}_{y1} +\cdots$.

The order $1 (= \epsilon ^{0})$ terms then correspond to the force-free case and at order $\epsilon ^{1}$ we obtain

29 $$\begin{aligned} \bar{\Phi}_{1} =& - \frac{2}{\left (b+\frac{1}{2}\right )\left (\frac{\beta _{i}}{\beta _{e}}+1\right )} \left [ \bar{A}_{x,ff} + \frac{\sinh (\bar{z})}{\cosh ^{2}(\bar{z})} \bar{A}_{x1} + \frac{1}{\cosh ^{2}(\bar{z})}\bar{A}_{y1}\right ], \end{aligned}$$

30 $$\begin{aligned} \frac{d^{2}\bar{A}_{x1}}{d\bar{z}^{2}} =& \left (1 - \frac{2}{\cosh ^{2}(\bar{z})}\right )\bar{A}_{x1} - \frac{\sinh (\bar{z})}{\cosh ^{2}(\bar{z})}\bar{\Phi}_{1}, \end{aligned}$$

31 $$\begin{aligned} \frac{d^{2}\bar{A}_{y1}}{d\bar{z}^{2}} =& - \frac{1}{\cosh ^{2}(\bar{z})}[2\bar{A}_{y1} + \bar{\Phi}_{1}]. \end{aligned}$$

Using Equation 29 on the right-hand sides of Equations 30 and 31 results in two coupled linear inhomogeneous second-order differential equations for $\bar{A}_{x1}$ and $\bar{A}_{y1}$. We emphasize that solving Equations 30 and 31 will result in electromagnetic potentials, and thus magnetic fields, that are self-consistent up to order $\epsilon $.

This system of equations has to be completed by initial and/or boundary conditions. The fact that the current density has to have a finite extent in $\bar{z}$ results in the requirement that $\bar{A}_{x1} \to 0$ as $|\bar{z}| \to \infty $. The reason for this is the first term on the right hand side of the $\bar{A}_{x1}$ equation. Because $\bar{A}_{x1}$ tends to a constant (here equal to zero) as $|\bar{z}| \to \infty $ we also ensure that $\bar{B}_{y1} \to 0$ in that limit and therefore $\bar{B}_{y} \to 0$ as well. For $\bar{A}_{y1}$ we impose the condition $\mathrm{d} \bar{A}_{y1}/d \bar{z} \to 0$ for $|\bar{z}| \to \infty $. This ensures that $\bar{B}_{x1} \to 0$ in this limit and that $\bar{B}_{x}$ approaches its force-free value outside the current sheet.

**Figure 1:**
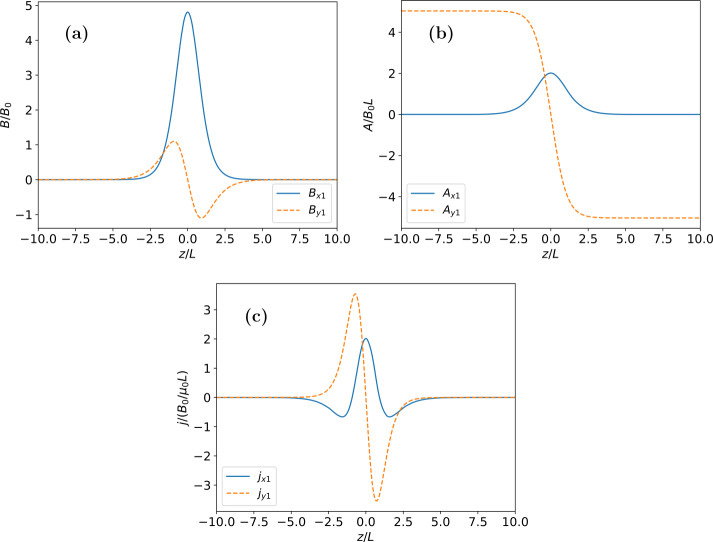
Solutions to the linear ODE system for the (a) magnetic field, (b) magnetic vector potential, and (c) current density components for the parameter values $b=0.9$, $T_{e}/T_{i}=1.0$, $\kappa _{e}=1.1$, and $\epsilon = 0.05$.

**Figure 2:**
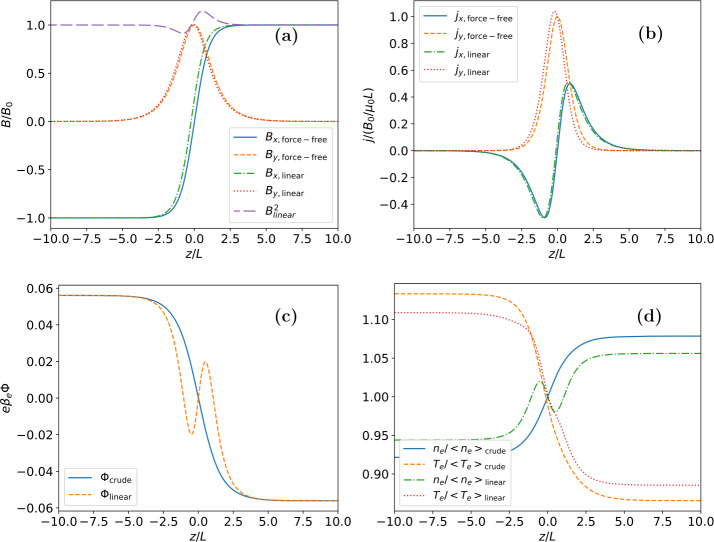
Resulting linear solutions of the (a) magnetic field profile, (b) current density profile, (c) electric potential, and (d) density and temperature asymmetries for the parameter values $b=0.9$, $T_{e}/T_{i}=1.0$, $\kappa _{e}=1.1$, and $\epsilon = 0.05$. Each panel compares the linear solution with the “crude” approximation for the same parameter values (see Section 3.2). The magnetic field and current density components for the “crude” approximation are those of the force-free solution.

**Figure 3:**
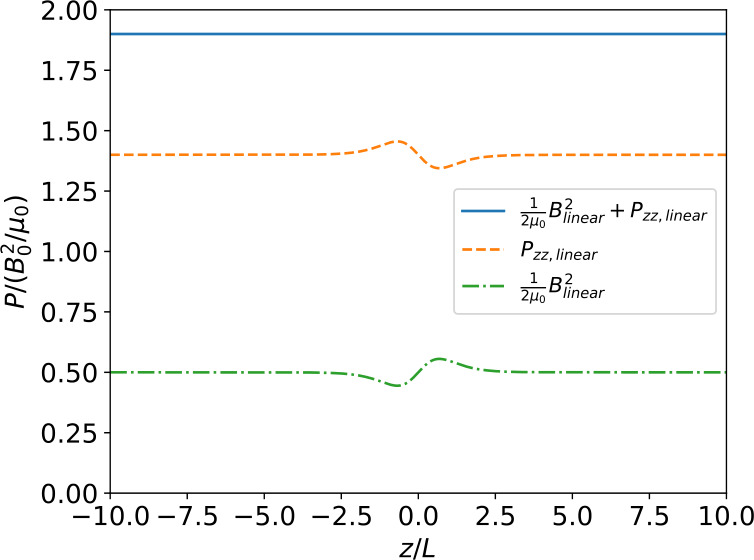
Variation with $z$ of the magnetic pressure and $zz$-component of the pressure tensor for the full linear solution, i.e. including all terms up to order $\epsilon $. Although both the magnetic pressure and the $zz$-component of the pressure tensor vary with $z$ their sum is constant, which shows that force balance holds up to order $\epsilon $ (for details on force balance see Appendix A).

Equations 29 – 31 were solved numerically for the same parameter values used in Neukirch et al. (2020), i.e. $b=0.9$ (which corresponds to a plasma $\beta$ of 1.4), $T_{e}/T_{i}=1.0$, $\kappa _{e}=1.1$, and $\epsilon =0.05$. The resulting solutions to the linear ODE system for the magnetic field, magnetic vector potential, and current density components are shown in Figure 1. The resulting profiles for the magnetic field, current density, electric potential, and particle density and temperature are shown in Figure 2. The two terms (magnetic pressure and $zz$-component of the pressure tensor) contributing to the force balance equation for the linear solution up to order $\epsilon $ are shown in Figure 3. As stated in Appendix A force balance is equivalent to the total pressure being constant with respect to the $z$-coordinate, and Figure 3 shows that this is case here. We remark that we have also calculated solutions for a number of other parameter combinations, but found that the solutions look very similar apart from very simple differences due to, for example, scaling of amplitudes.

The magnetic field and current density profiles are broadly consistent with those of the force-free case (e.g. Harrison and Neukirch 2009a; Neukirch, Wilson, and Harrison 2009) with a small shift of the new profiles in $z$ noticable. This is caused by the additional terms in the current density and the need for the first order quantities to satisfy the imposed boundary and initial conditions. As a consequence of this the magnetic field is no longer completely force-free, as can be seen by the deviation of $B^{2}(\bar{z})$ from a straight line within the current sheet. The amplitude of this deviation is, however, small.

Somewhat surprisingly, the electric potential and the electron density and temperature profiles display additional spatial structures around the centre of the sheet which are different from those found under the assumption that the magnetic field is unchanged at first order in $\epsilon $. Hence, the inclusion of $A_{x}$ and $A_{y}$ in the expansion leads to particle density profiles that are not simply asymmetrical, but also have some spatial substructure within the current sheet.

### Nonlinear Theory

To corroborate the results found by using the expansion method, we also solved the full nonlinear problem as formulated in Equations 13 – 16. Due to the nonlinear nature of this differential algebraic system of equations (DAE) solutions must be found numerically.

The normalised quasineutrality condition given by Equation 25 is a transcendental equation and cannot be solved analytically for $\bar{\Phi}$. Hence, one needs to solve the full set of equations given by Equations 13 – 16. To do so, we follow the method used by Catapano et al. (2015). This method replaces the algebraic quasineutrality condition (Equation 25) by a differential equation for the electric potential $\bar{\Phi}$. This differential equation is obtained by simply differentiating the quasineutrality condition with respect to $\bar{z}$, leading to the ODE

32 $$\begin{aligned} \frac{d \bar{\Phi}}{d\bar{z}} =& \left (\left [e^{\bar{\Phi}}\sin (2 \bar{A}_{x})+2\epsilon (1+\kappa _{e}\bar{\Phi})e^{\kappa _{e} \bar{\Phi}}\right ]\frac{d \bar{A}_{x}}{d \bar{z}} +\left [2e^{ \bar{\Phi}}e^{2\bar{A}_{y}}\right ]\frac{d \bar{A}_{y}}{d \bar{z}} \right ) \\ & \hspace{2em} \cdot \left (-\left [b+\frac{1}{2}\right ] \frac{\beta _{i}}{\beta _{e}}e^{-\frac{\beta _{i}}{\beta _{e}} \bar{\Phi}} -e^{\bar{\Phi}}N(\bar{A}_{x},\bar{A}_{y}) \right . \\ & \hspace{12em} \left . -2\epsilon \kappa _{e}\bar{A}_{x}(2+\kappa _{e}\bar{\Phi})e^{ \kappa _{e}\bar{\Phi}}\right )^{-1} . \end{aligned}$$

Equation 32 is solved alongside Equations 27 and 28 to obtain a solution to the full nonlinear problem. To facilitate the use of similar boundary conditions as in the linear case, we define the differences $\Delta \bar{A}_{x}$ and $\Delta \bar{A}_{y}$ between the vector potential components and their force-free counterparts by $\bar{A}_{x} = \bar{A}_{x,ff} + \Delta \bar{A}_{x}$, $\bar{A}_{y} = \bar{A}_{y,ff} + \Delta \bar{A}_{y}$, and reformulate the set of differential equations for this difference (Appendix B). The boundary conditions used for the nonlinear calculations are $\mathrm{d} \Delta \bar{A}_{x} /\mathrm{d} \bar{z} \to 0$ and $\mathrm{d} \Delta \bar{A}_{y} /\mathrm{d} \bar{z} \to 0$ for $\left |\bar{z}\right | \to \infty $, which are similar to those of the linear case and ensure that one obtains a spatially confined current density structure, i.e. a current sheet. Due to the additional differential equation for the electric potential one has to impose a fifth condition, which has been chosen as $\Delta \bar{A}_{y} \to 0 $ for $\bar{z} \to - \infty $.

**Figure 4:**
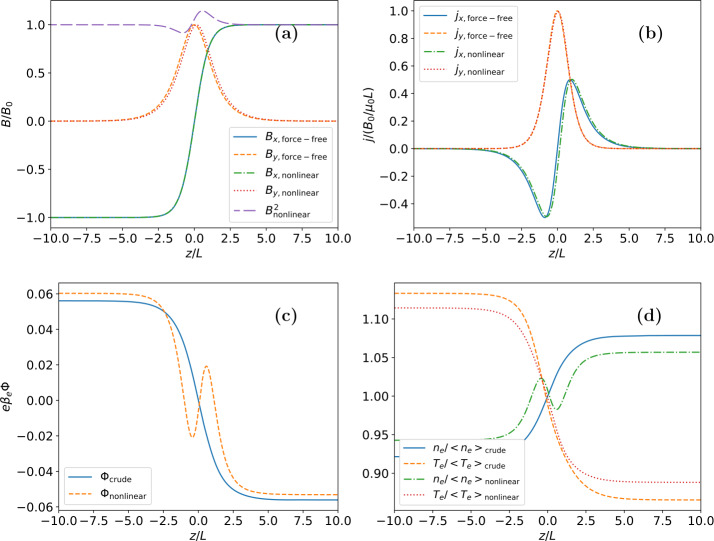
Resulting nonlinear solutions of the (a) magnetic field profile, (b) current density profile, (c) electric potential, and (d) density and temperature asymmetries for the parameter values $b=0.9$, $T_{e}/T_{i}=1.0$, $\kappa _{e}=1.1$, and $\epsilon = 0.05$. Each panel compares the nonlinear solution with the “crude” approximation (Section 3.2). The magnetic field and current density components for the “crude” approximation case are those of the force-free solution.

**Figure 5:**
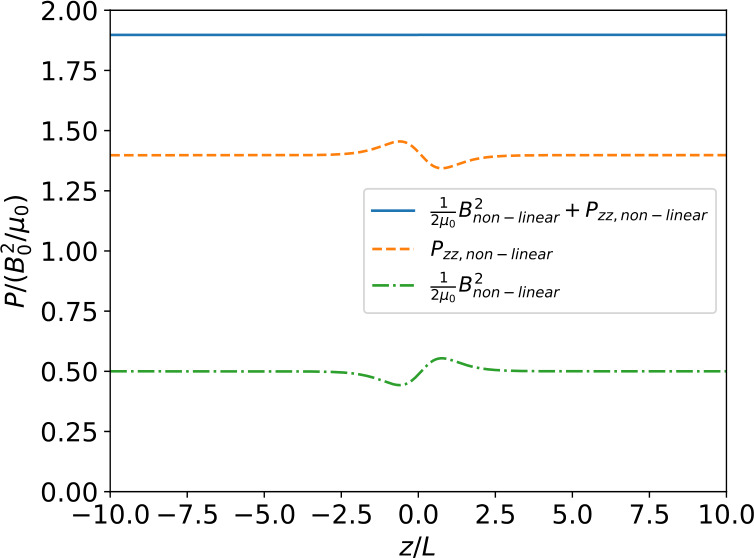
Variation with $z$ of the magnetic pressure, $zz$-component of the pressure tensor and their sum (total pressure) for the full non-linear solution. Both the magnetic pressure and the $zz$-component of the pressure tensor vary, but the total pressure is constant implying that the current sheet is in force balance (for details on force balance see Appendix A).

We show in Figure 4 the results of the numerical calculation for the self-consistent magnetic field, current density, electric potential, and particle density and temperature. In Figure 5 we show the variation across the current sheet of the magnetic pressure, the $zz$-component of the pressure tensor and their sum (total pressure) for the full non-linear problem. As Figure 5 shows the total pressure is constant across the sheet which implies that the current sheet is in force balance.

In these calculations the same parameter values as in Figures 1 – 3 (${b=0.9}$, $T_{e}/T_{i}=1.0$, $\kappa _{e}=1.1$, $\epsilon =0.05$) have been used. Solutions for a number of other parameter combinations have also been calculated, but were found to be structurally very similar, albeit with somewhat different amplitudes, for example.

As in the linear case, the magnetic field and current density profiles do not deviate much from the force-free case (Harrison and Neukirch 2009a; Neukirch, Wilson, and Harrison 2009) although they again are slighty different, which is due to the additional dependence of the current density on the electric potential in combination with the imposed boundary conditions. Both the magnetic field and current density variations are largely identical for the linear and the nonlinear cases, showing the consistency of the results for both cases.

One also sees that the electric potential and electron density and temperature profiles show spatial variations very similar to those found in the linear case. The nonlinear calculations are thus fully consistent with the results of the linear case and corroborate these. The consistency between the linear expansion method and the nonlinear case also shows that the “crude” approximation fails to provide the correct solution for the electric potential and the particle density. In view of the relatively minor differences between the force-free magnetic field and the magnetic fields found in the linear expansion method and the nonlinear case, this is a somewhat surprising result, but shows that making consistent assumptions is important.

## Summary and Conclusions

In this article we have presented a generalisation of the theoretical approach by Neukirch et al. (2020) to model observed systematic asymmetries of particle density and temperature across kinetic scale solar wind current sheets. In the previous approach, both ion and electron distribution functions were modified from a known force-free case (e.g. Harrison and Neukirch 2009a; Neukirch, Wilson, and Harrison 2009) to maintain exact charge neutrality leading to a vanishing electric potential. Here we have investigated the consequences of modifying only the electron distribution function. This leads to a system of equations in which a nonlinear quasineutrality condition coupling the electric potential and the vector potential has to be solved alongside Ampère’s law. We showed that a “crude” approximation method in which only the electric potential is expanded in terms of a small parameter, while the force-free magnetic field is kept unchanged (Neukirch et al. 2022), is inconsistent. We have presented results of both a consistent expansion method which includes the magnetic field (up to linear terms) and of solving the full nonlinear problem. These results showed that while the changes in the magnetic field are relatively small, the quasineutral electric potential has an unexpected spatial substructure inside the current sheet, which in turn leads to a similar spatial substructure of the particle density.

While this is an interesting theoretical result, it is different from the monotonically varying particle density (and temperature) profiles found by Neukirch et al. (2020). One immediate question that arises is whether there are any observational examples at all that display a spatial substructure of a similar nature to our findings in this article. We do not claim that the following constitutes a proper comparison of our theory with the observations. However, there seem to be examples of current sheet observations in the solar wind that display substructures not too dissimilar to those found in this study.

**Figure 6:**
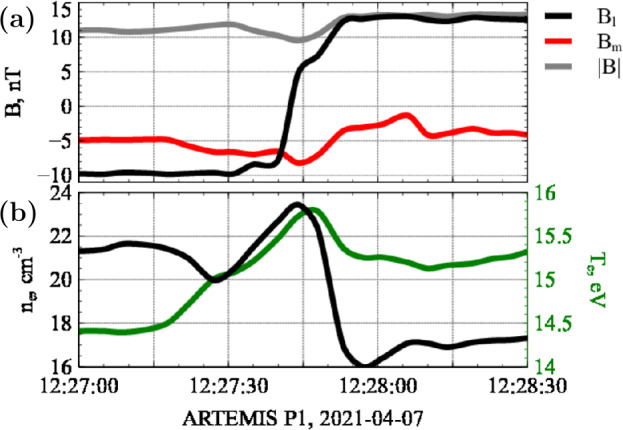
An example of a current sheet observed by the Artenis mission near 1 AU (Angelopoulos 2011) showing (a) the magnetic field components in the local coordinate system, and (b) the plasma (electron) density and electron temperature profiles. We remark that the theoretical result presented before shows spatial variations of the electron density in the opposite direction to the observation shown here but that is merely a consequence of the symmetry chosen for the theoretical calculations which could be reversed without problem.

Figure 6 shows one such example observed by the Artemis mission near 1 AU (Angelopoulos 2011). The top panel presents the magnetic field measurements from the fluxgate magnetometer (Auster et al. 2008). A local coordinate system is used, with $B_{l}\approx B_{x}$ and $B_{m} \approx B_{y}$ (for details on current sheet selection and magnetic field processing, see Artemyev, Angelopoulos, and Vasko 2019). The magnetic field configuration exhibits a $B_{l}$ reversal and a peak in $|B_{m}|$, indicative of a nearly constant magnetic field magnitude ($B \approx const$) across a force-free current sheet. Since solar wind current sheets are convected by plasma flows, the time series can be interpreted in terms of spatial scales (see, e.g. Artemyev, Angelopoulos, and Vasko 2019; Neukirch et al. 2020). The bottom panel shows the plasma (electron) density and electron temperature profiles. In this particular current sheet, the ion temperature is approximately half that of the electron temperature. Superimposed on the general trends—an increase in density and a decrease in temperature—are non-monotonic substructures near the current sheet center (at the $B_{l}$ reversal), which resemble those predicted by the theoretical model. Although a more detailed analysis of plasma kinetics, including the velocity distribution functions and their evolution across the current sheet, is necessary for a quantitative comparison, the example in Figure 6 illustrates the potential for such observational–theoretical correspondence.

We have not carried out a full systematic comparison here, and our results are not the only possible explanation of the observations shown. In particular it will be important to assess the full details of pressure balance in future work (beyond the scope of the work presented here). However, it is important to note the observations of current sheets in the solar wind that have a similar substructure to our theoretical findings.

## Figures and Tables

**Figure 7:**
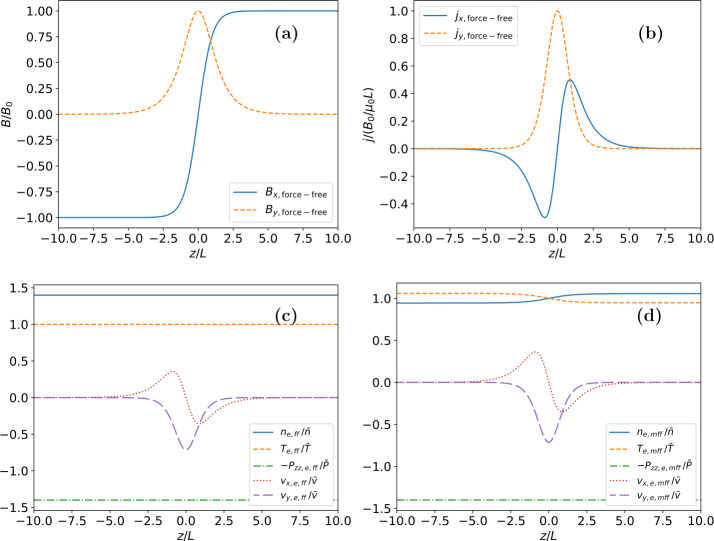
Profiles of the force-free (a) magnetic field and (b) current density, and the electron density, temperature, pressure, and drift velocity for the (c) force-free case (labelled ‘ff’ in the legend) and (d) modified force-free case (labelled ‘mff’ in the legend). Note that $-P_{zz,e}$ has been plotted since $P_{zz,e}$ has the same profile as $n_{e}$ in the force-free case. We also note that the normalising quantities in panels c and d are as follows: $\bar{n}=n_{0}(b+1/2)$, $\bar{P}=B_{0}^{2}/\mu _{0}$, $\bar{T}=B_{0}^{2}/(k_{B}\mu _{0}n_{0}(b+1/2))$, and $\bar{v}=\left |u_{0}\right |/(b+1/2)$.

## Data Availability

Artemis data are available at http://themis.ssl.berkeley.edu. Data was retrieved and analyzed using SPEDAS, see Angelopoulos et al. (2019).

## Appendix A: Basic Current Sheet Models: Further Details

For reference, we briefly summarise in this appendix further details of one-dimensional collisionless current sheet equilibrium theory. For the coordinate system chosen in this article a crucial quantity is the $zz$-component of the plasma pressure tensor, which is defined as

33 $$ P_{zz}(\Phi , A_{x}, A_{y})= \sum _{s} m_{s} \int v_{z}^{2} F_{s}(H_{s}, p_{x,s}, p_{y,s})\, \mathrm{d}^{3} v. $$

It can be shown (see, e.g. Mynick, Sharp, and Kaufman 1979; Harrison and Neukirch 2009b) that

34 $$\begin{aligned} \sigma =& -\frac{\partial P_{zz}}{\partial \Phi} , \end{aligned}$$

35 $$\begin{aligned} j_{x} =& \frac{\partial P_{zz}}{\partial A_{x}} , \end{aligned}$$

36 $$\begin{aligned} j_{y} =& \frac{\partial P_{zz}}{\partial A_{y}} , \end{aligned}$$

which makes it very convenient to use $P_{zz}$ to determine the charge density, $\sigma $, and the components of the current density, $j_{x}$ and $j_{y}$. For the assumptions made in this article, a self-consistent one-dimensional quasineutral collisionless current sheet automatically satisfies the force balance equation, which is the first order velocity moment of the stationary Vlasov equation (see, e.g. Mynick, Sharp, and Kaufman 1979). The only non-trivial component of the force balance equation is the $z$-component, given by

37 $$ \frac{\mathrm{d}}{\mathrm{d} z}\left ( \frac{B_{x}^{2} +B_{y}^{2}}{2\mu _{0}} + P_{zz}\right ) =0. $$

For a one-dimensional force-free current sheet the force balance equation splits into the two conditions (see, e.g. Harrison and Neukirch 2009b)

38 $$\begin{aligned} \frac{\mathrm{d}}{\mathrm{d} z}\left ( \frac{B_{x}^{2} +B_{y}^{2}}{2\mu _{0}} \right ) =& 0, \end{aligned}$$

39 $$\begin{aligned} \frac{\mathrm{d} P_{zz}}{\mathrm{d} z} =& 0, \end{aligned}$$

implying that both $B^{2}$ and $P_{zz}$ are constant for a one-dimensional force-free current sheet.

The contributions to the $zz$-component of the pressure tensor resulting from the electron distribution function given by Equation 7 and the ion distribution function given by Equation 8, respectively, are

40 $$\begin{aligned} P_{e,ff} =& \frac{n_{0}}{\beta _{e}} e^{e\beta _{e}\Phi} N(A_{x},A_{y}), \end{aligned}$$

41 $$\begin{aligned} P_{i,ff} =& \frac{n_{0,i}}{\beta _{i}} e^{-e\beta _{i}\Phi} , \end{aligned}$$

where $N(A_{x},A_{y})$ is defined in Equation 14. The derivatives with respect to $A_{x}$ and $A_{y}$ of $P_{e,ff}$ ($P_{i,ff}$ does not depend on the vector potential) result in the first terms on the right hand sides of Ampère’s law for Equations 15 and 16 for $\epsilon =0$. Setting the derivative with respect to $\Phi $ equal to zero results in the quasineutrality condition for the normal force-free case

42 $$ n_{0,i} e^{-e\beta _{i}\Phi} - n_{0} e^{e\beta _{e}\Phi} N(A_{x},A_{y}) = 0 $$

Using standard identities it is straightforward to show that $N(A_{x,ff}, A_{y,ff}) = b+1/2$ (Equation 17) (e.g. Harrison and Neukirch 2009a; Neukirch, Wilson, and Harrison 2009; Neukirch, Wilson, and Allanson 2018) and that therefore Equation 42 is solved by $\Phi = 0$, with $n_{0,i} = n_{0}(b+ 1/2)$ (Equation 18).

The current density resulting from the force-free magnetic field in Equation 6 is

43 $$ \mu _{0} \mathbf{j}_{ff}(z) = \frac{B_{0}}{L \cosh ^{2}(z/L)} [\sinh (z/L), 1, 0]. $$

Plots of the force-free magnetic field and current density components as well as the electron density, $zz$-component of the electron pressure tensor, electron temperature and electron drift velocity (for both force-free and the modified force-free case) are shown in Figure 7. For comparison, we have also included plots of the magnetic field and current density in Figures 2 (panels a and b, respectively) and 4 (panels a and b). Again, one can easily verify analytically that $j_{x,ff}(z) = en_{0}u_{0}\frac{1}{2}\sin (e\beta _{e}u_{0}A_{x,ff})$ and $j_{y,ff}(z) = en_{0}u_{0}e^{-e\beta _{e}u_{0}A_{y,ff}}$ if the relations given by Equations 19 – 22 hold (e.g. Harrison and Neukirch 2009a; Neukirch, Wilson, and Harrison 2009; Neukirch, Wilson, and Allanson 2018).

The additional distribution function given by Equation 11 results in additional terms for the $zz$-component of the pressure tensor for particle species $s$ of the form

44 $$ P_{\Delta ,s} = - \delta n_{s} q_{s}^{2} \beta _{s} u_{0} A_{x} \Phi e^{- \kappa _{s} \beta _{s} q_{s} \Phi}. $$

The additional terms for the charge density and hence the quasineutrality condition can be calculated using Equation 34. Using the relation given by Equation 19 and $\delta n_{e} = - \delta n_{i} \beta _{i}/\beta _{e}= \epsilon n_{0}$, one obtains

45 $$\begin{aligned} \Delta \sigma _{e} = &- e \epsilon n_{0} \frac{2 A_{x}}{B_{0} L} (1 + \kappa _{e} \beta _{e} e \Phi ) e^{\kappa _{e} \beta _{e} e \Phi}, \end{aligned}$$

46 $$\begin{aligned} \Delta \sigma _{i} =& e \epsilon n_{0} \frac{2 A_{x}}{B_{0} L} \left (1 - \kappa _{i} \beta _{i} e \Phi \right ) e^{-\kappa _{i} \beta _{i} e \Phi}. \end{aligned}$$

It is obvious that $\Phi =0 $ is still a solution of the quasineutrality equation even if the term $\Delta \sigma _{e} + \Delta \sigma _{i}$ is added.

Because the additional pressure tensor term only depends on $A_{x}$ according to Equation 35 it will only contribute to $j_{x}$. The partial derivative with respect to $A_{x}$ of $P_{\Delta ,s}$ is given by

47 $$ \frac{\partial P_{\Delta ,s}}{\partial A_{x}} = - q_{s} \delta n_{s} u_{0} (\beta _{s} q_{s} \Phi ) e^{-\kappa _{s} \beta _{s} q_{s} \Phi }, $$

which vanishes for $\Phi =0$. Hence for the case in which both the electron and the ion distribution functions are modified the current density remains unchanged and hence the force-free magnetic field stated in Equation 6 remains a self-consistent solution of the modified problem.

In all of the cases discussed before (purely force-free and modified force-free) or in this article the electric current density is carried by the electrons only, which also implies that only the electrons have a non-vanishing average velocity, which is identical to the current density divided by the electron charge density. Like all quantities the average velocity only depends on $z$, and since only its $x$- and $y$-components are non-zero it does not contribute in any way to the force balance of the system.

## Appendix B: Reformulated Nonlinear Differential Equations

Using the differences $\Delta \bar{A}_{x}$ and $\Delta \bar{A}_{y}$ between the vector potential components and their force-free counterparts defined by $\bar{A}_{x} = \bar{A}_{x,ff} + \Delta \bar{A}_{x}$, $\bar{A}_{y} = \bar{A}_{y,ff} + \Delta \bar{A}_{y}$, Equations 27, 28 and 32 are reformulated as

48 $$\begin{aligned} \frac{d^{2}\Delta \bar{A}_{x}}{d\bar{z}^{2}} =& \frac{\sinh (\bar{z})}{\cosh ^{2}(\bar{z})}\left (-e^{\bar{\Phi}} \cos (2\Delta \bar{A}_{x}) + 1\right ) - \frac{2 - \cosh ^{2}(\bar{z})}{2\cosh ^{2}(\bar{z})}e^{\bar{\Phi}} \sin (2\Delta \bar{A}_{x}) \\ & \hspace{20em} - \epsilon \bar{\Phi}e^{\kappa _{e}\bar{\Phi}}, \end{aligned}$$

49 $$\begin{aligned} \frac{d^{2}\Delta \bar{A}_{y}}{d\bar{z}^{2}} =& \frac{1}{\cosh ^{2}(\bar{z})}\left (-e^{\bar{\Phi}}e^{2\Delta \bar{A}_{y}} + 1\right ), \end{aligned}$$

50 $$\begin{aligned} \frac{d\bar{\Phi}}{d\bar{z}} =& \biggl\{ \biggl( \frac{2\sinh (\bar{z})}{\cosh ^{2}(\bar{z})}e^{\bar{\Phi}}\cos (2 \Delta \bar{A}_{x}) + \frac{2-\cosh ^{2}(\bar{z})}{\cosh ^{2}(\bar{z})}e^{\bar{\Phi}}\sin (2 \Delta \bar{A}_{x}) \\ & \hspace{1.5em} +2\epsilon (1+\kappa _{e}\bar{\Phi})e^{\kappa _{e}\bar{\Phi}}\biggr) \cdot \left (\frac{1}{\cosh (\bar{z})}+ \frac{d\Delta \bar{A}_{x}}{d\bar{z}}\right ) \\ & \hspace{2.5em} + \left (\frac{2}{\cosh ^{2}(\bar{z})}e^{\bar{\Phi}}e^{2\Delta \bar{A}_{y}}\right ) \cdot \left (- \frac{\sinh (\bar{z})}{\cosh (\bar{z})}+ \frac{d\Delta \bar{A}_{y}}{d\bar{z}}\right )\biggr\} \\ & \hspace{3.5em} \cdot \biggl\{ -\frac{\beta _{i}}{\beta _{e}}\left (b+\frac{1}{2} \right )e^{-\frac{\beta _{i}}{\beta _{e}}\bar{\Phi}} -e^{\bar{\Phi}} \left ( \frac{\sinh (\bar{z})}{\cosh ^{2}(\bar{z})}\sin (2\Delta \bar{A}_{x})\right . \\ & \hspace{4.5em} \left . - \frac{2-\cosh ^{2}(\bar{z})}{2\cosh ^{2}(\bar{z})}\cos (2 \Delta \bar{A}_{x}) + \frac{1}{\cosh ^{2}(\bar{z})}e^{2\Delta \bar{A}_{y}} +b\right ) \\ & \hspace{5.5em} -2\epsilon \kappa _{e}(2+\kappa _{e}\bar{\Phi})e^{\kappa _{e} \bar{\Phi}}(\bar{A}_{x,ff}+\Delta \bar{A}_{x})\biggr\} ^{-1}. \end{aligned}$$
